# Prevalence of vitamin B complex deficiencies in women in reproductive age, pregnant, or lactating woman in Brazil: a systematic review and meta-analysis protocol

**DOI:** 10.1186/s13643-022-02136-7

**Published:** 2023-01-25

**Authors:** Michel Carlos Mocellin, Cintia Chaves Curioni, Alessandra da Silva Pereira, Simone Augusta Ribas, Michelle Teixeira Teixeira, Tatiane Salgado Galvão de Macedo, Gabriel Mantolvão Palermo

**Affiliations:** 1 Department of Fundamental Nutrition, Nutrition School, Federal University of the State do Rio de Janeiro, Rio de Janeiro, Brazil; 2 Department of Social Nutrition, Institute of Nutrition, State University of Rio de Janeiro, Rio de Janeiro, Brazil; 3 Department of Public Health Nutrition, Nutrition School, Federal University of the State do Rio de Janeiro, Rio de Janeiro, Brazil; 4 Institute of Nutrition, State University of Rio de Janeiro, Rio de Janeiro, Brazil; 5 Nutrition School, Federal University of the State of Rio de Janeiro, Rio de Janeiro, Brazil

**Keywords:** Systematic review, Women, Vitamins B complex, Fertility, Childbearing age, Fertile period, Pregnancy, Lactation, Brazil, Nutritional deficiency

## Abstract

**Background:**

Vitamin B deficiencies are involved with several outcomes in fertility and pregnancy. In Brazil, the national prevalence rates of these micronutrient deficiencies in women of reproductive age were not known. This study aims to systematically identify, select, evaluate, analyze, and report the prevalence rates of vitamin B complex deficiencies in women of reproductive age in Brazil and identify variables that may modify the outcome rates.

**Methods:**

A systematic review will be conducted guided by the following question: “What is the prevalence of vitamin B deficiencies in women of reproductive age in Brazil?”. The studies will be identified and selected from a literature search using electronic databases, consultation with researchers/specialists, and reference lists of eligible studies and reviews on the topic. Major eligibility criteria include observational cross-sectional and cohort studies carried out in Brazil and performed in women 10–49 years old, or pregnant and lactating mothers, and investigated the deficiency of vitamin B complex by laboratory test. Two reviewers independently will perform the screening and selection of the studies, data extraction, and risk of bias assessment. For the data report, a narrative approach will be used to present the characteristics of the included studies and individual findings. A random meta-analysis model will be implemented to summarize the individual prevalence rates in a global value if the studies are sufficiently homogeneous.

**Discussion:**

This study aims to identify the national and regional prevalence rates of vitamin B complex deficiencies in women of reproductive age; allow the policymakers discuss, plan, and implement public policies to screen; and prevent and/or treat these malnutrition conditions. This also aims to know the rates of nutritional deficiencies over the years, serving as an indirect indicator of the socioeconomic and dietary patterns of the population. Specifically for folate, this study allows to compare the prevalence rates of deficiency of this vitamin before and after the mandatory fortification of wheat and corn flours implemented since 2004 in Brazil, in this specific population. The evidence gathered may highlight the need for population-based studies to investigate the deficiency of these vitamins.

**Systematic review registration:**

PROSPERO CRD42020188474

**Supplementary Information:**

The online version contains supplementary material available at 10.1186/s13643-022-02136-7.

## Background

Almost two billion people have micronutrient deficiencies, affecting mainly women of reproductive age, pregnant women, and children in low- and middle-income countries [1, 2]. Among the possible causes are poor diet quality, characterized by high consumption of ultra-processed foods, rich in saturated fat, sugar, and sodium [3], and the presence of infections and/or chronic diseases that can compromise food intake and nutrient absorption [4]. In Brazil, a sum of variables turns their population at the risk for nutritional deficiencies, such as low purchasing power, regions with unfavorable environmental conditions for food production and soil poor in some essential nutrients, unhealthy food habits, and high prevalence rates of chronic diseases [5].

In the female population, the continuous use of oral contraceptives can contribute to triggering the deficiency of micronutrients such as folic acid; Riboflavin, vitamins B6, B12, C and E; and the minerals magnesium, selenium, and zinc [6, 7]. In turn, the periods of pregnancy and lactation are characterized by high nutritional demand, specifically the vitamin B complex [8, 9], making it more challenging to maintain nutritional adequacy [10]. Low concentrations of these vitamins have been associated with pregnancy complications such as neural tube defects [11, 12] and spontaneous abortion [12].

Vitamin B complex are involved in the one-carbon metabolic pathway responsible for homocysteine (Hcy) metabolism and the formation of S-adenosylmethionine (SAM), which serves as the main methyl donor for methylation, collaborating in an epigenetic mechanism to regulate gene expression and is essential for normal embryonic development [13, 14]. Another important action of these vitamins is to serve as a cofactor for enzymatic activity in the metabolism of carbohydrates, fats, and proteins.

Dietary deficiency of vitamins B6, B12 and folate or genetic alterations in the folate activation pathway can lead to Hcy accumulation [15]. In high concentrations, Hcy is pro-inflammatory, leads to oxidative stress [14, 15], and has been identified as an independent risk factor for atherosclerotic vascular disease [16, 17] and is associated with infertility [18] and adverse pregnancy outcomes [11], such as pre-eclampsia [19, 20] and preterm birth [21].

In order to decrease the prevalence of micronutrient deficiencies that mainly affect the maternal-infant group, public health policies have been implemented for decades, such as fortification of wheat and maize flours [22], and the universal folic acid supplementation for women who wish to become pregnant and for all pregnant women until the end of pregnancy recommended by WHO [23] and the Brazilian Ministry of Health [24].

Despite all efforts so far implemented, monitoring the effectiveness of micronutrient supplementation or fortification interventions, as well as the evolution of cases of deficiency, is still a challenge in the Brazil, due to the scarcity of screening studies of epidemiological and population-based nutrient deficiency rates. It is added that the heterogeneity that characterizes the Brazilian population, either in relation to the socioeconomic and cultural conditions, reinforces the importance of identifying the real number of cases of micronutrient deficiencies that affect women of fertile age, pregnant, and lactating women in the country.

In view of these considerations, we designed this systematic review protocol to answer the following research question: What is the prevalence of vitamin B complex deficiency in women at reproductive age in Brazil? This research can contribute to the Brazilian National Food and Nutrition Policy designed in 1999 [25], more precisely, with its guidelines of Food and Nutrition Surveillance; Research, Innovation and Knowledge in Food and Nutrition; and Management of Food and Nutrition Actions; besides National Policy for Integral Attention to Women’s Health - 2004 [26] and the National Policy Plan for Women 2013–2015 [27] in order to present an epidemiological scenario on the subject to guide and evaluate the actions implemented so far.

### Objective

This review will systematically examine, identify, select, evaluate, and analyze all studies that report the prevalence rates of vitamin B complex deficiencies in Brazilian women at fertile age, pregnant, or in lactation, and identify possible variables that may modify the outcome rates.

## Methods and design

The review protocol follows the recommendations of the Joanna Briggs Institute Manual of Evidence Synthesis [28]. In addition, to ensure transparency and non-duplicity of the publication, as well as to minimize the presence of biases during its execution, this protocol was publicly registered in the PROSPERO database (CRD42020188474). The present study protocol is being reported in accordance with the reporting guidance provided in the Preferred Reporting Items for Systematic Reviews and Meta-Analyses Protocols (PRISMA-P) statement [29].

### Review question

What is the prevalence of vitamin B complex deficiency in women of reproductive age in Brazil?

### Eligibility criteria

Studies will be selected according to the following criteria:

#### Population/participants

The participants are women of reproductive age (10 to 49 years of age), including pregnant and lactating mothers.

#### Outcome(s)/clinical situation of interest

The outcome of interest in this review is the deficiency of any vitamins from the B complex, diagnosed by laboratory/biochemical analysis following the recommended methods. No cut-off values were taken as an inclusion criterion. Studies in which the deficiency was identified by clinical signals and symptoms will not be eligible.

### Study design

This systematic review will include cross-sectional studies or cohorts. In case of a cohort with multiple investigations of our interesting outcome, in global analysis, we will use the rate of the most recent analysis in which it was investigated, but for temporal analysis, all rates investigated in distinct moments of the follow-up will be included. Only fully published studies in the form of a scientific article or academic thesis, dissertation, or monograph will be eligible. No published studies or studies without full-text access, besides conference proceedings, editorials, case reports, case-control, letters, and trials will be not eligible.

#### Epidemiologic indicator

Studies that provided the prevalence rates of the outcome will be eligible. Original studies that did not present the prevalence rates but described the proportion of eligible women with deficiency of any vitamin from B complex will be eligible if the prevalence may be calculated.

#### Setting/location

Only studies carried out in Brazil will be eligible. Studies in any setting (primary care, hospital, outpatient, clinic patients) representing the general population or subgroups that reflect populational conditions will be eligible. It will be ineligible: studies carried out only with patients with diseases or any pathological condition (except obesity associated with their comorbidities); situations that affect the digestion, absorption, metabolization rates, and daily requirements of the vitamins such as post-bariatric surgery, athletes, and vegetarians; and studies on which the vitamins deficiencies are assessed in a woman with genetic polymorphisms/variants that affect the body status of the vitamin B complex. However, studies that determine the prevalence of the polymorphism related to and the deficiency rates of vitamin B, not conditioning one to another, will be eligible.

### Information sources and search strategy

The source of literature will be a systematic and structured search of the following electronic databases, planned to take place in 2020, June, and updated in 2022, June: MEDLINE (PubMed), Embase, Web of Science (main collection), Scopus, SciELO Citation Index (by Web of Science), *Literatura Latino Americana e do Caribe em Ciências da Saúde* (by Biblioteca Virtual em Saúde - BIREME), and a Brazilian database of academic thesis and dissertations (Biblioteca Digital Brasileira de Teses e Dissertações - BDTD). In addition, we will be performing a consultation with Brazilian researchers in the areas of health, medicine, nutrition, and public health allocated in public universities of the country, by e-mail, requesting potentially eligible studies after the explanation of the objectives and inclusion criteria. Hand-searching of reference lists of included studies, relevant reviews, or other relevant documents will also be performed to search for potentially eligible studies. The literature searches in electronic databases were designed using search terms underpinning the inclusion criteria and ensured by MeSH Terms and Emtree terms. The search strategies were created by combining search terms with Boolean operators and using search facilitators (quotation marks, truncation symbol, proximity operators) and were adapted by each database. Before the official electronic search, the search strategies were piloted to ensure sufficient specificity and sensitivity. The search strategies that will be used in the official literature search are presented in Supplementary file 1. No limits on publication date and languages will be defined.

### Study selection

The results from the literature search will be transferred to Ryyan QCRI, the Systematic Reviews web app [30] for the management of the references. In the app, the repeated references will be identified by one reviewer and only one of its multiple records will remain for the next step. In the phase of study selection, two reviewers will be analyzing the titles, abstracts, and full texts for eligibility. They will be blinded, and at the conclusion of the process, the judgment of each will be confronted. In case of discrepant judgment about the study eligibility, this study will be analyzed by the reviewers, and a consensus will be reached by discussion. Initially, the titles will be read, and if it appears eligible, the abstracts and full texts will be read to confirm eligibility.

### Data extraction

Data extraction of the eligible studies will be performed by two reviewers, independently, registering the data in a customized Microsoft Office Excel® sheet, that will be pilot-tested prior to initiating the data extraction process. The extracted data by the two reviewers will be confronted and any disagreement resolved by new consultation of the study. In this process, the original authors may be consulted for any additional information not (fully) presented in the publication by e-mail or by Research Gate®. The information that will be extracted from the studies is the study identification, year of publication, type of publication, local (city and state), and period in which the study was conducted, study design, population group (pregnant, lactating woman, or women not pregnant or lactating), and their characteristics (age range or mean/median, setting of recruitment, the proportion of obese or malnourished participants, and the proportion of chronic diseases; the use of medicines and vitamin supplements; and other clinical and demographic characteristics; for pregnant, trimester of the analysis), sampling process, the number of participants recruited and analyzed, sample size calculation, vitamin B complex deficiencies investigated and their diagnostic method, the prevalence data, and their 95% CI or the number of cases for prevalence calculation.

### Data synthesis

Initially, we will perform a narrative description of the summary of the results. For the selection process, the results will be presented in accordance with the PRISMA flow diagram [31]. The information extracted about each study (characteristics) and the prevalence rates (and 95% IC) of vitamin B complex deficiencies will be presented in a summary table. The results will be presented separately for pregnant, lactating women, and women not pregnant or no lactating and for each vitamin B assessed.

Meta-analysis will be done using a random model and using STATA 16.0 software and the Metaprop and Metapreg packages. Random effects model will be applied to estimate the pooled prevalence since we will expect heterogeneity among the studies a priori. The proportion (prevalence) of cases will be transformed to logit before combining the data in the metanalytic approach. Statistics for heterogeneity (test *Q* de Cochran and *I*^2^) and sensitivity analysis will be also done. For the meta-analysis, we entered the software the proportion of cases and the total numbers o evaluated subjects.

For each subpopulation (pregnant, lactating women, and women not pregnant or no lactating) and each vitamin B, if there are two or more studies that fulfilled each subgroup criteria, we intend to stratify the analysis by the following:

- Time/year (temporal analysis), showing the prevalence rates in a timeline graph, and specific for folate, we will stratify in before 2004 and after 2004, because this year, it became mandatory to enrich wheat and corn flours with folic acid in the country
- Age group: 15 to 19 years; 20 to 35 years; >35 years
- State and Geographic Region of Brazil: 24 states + federal district; 5 macro-regions of the country (north, south, southeast; midwest; and northeast)
- For pregnant: trimester of gestation (1st, 2nd, or 3th)
- Lactation period: 1st semester, 2nd semester, and 2nd year
- Other that may reduce the possible heterogeneity among the studies and/or modify the prevalence rates. Individual meta-analysis will be done by each subgroup above described, including only studies that attend these subgroup criteria. Meta-regression may be applied to investigate these factors/variables associated with the modification of the prevalence rates.

### Risk of bias

The risk of bias will be assessed following the JBI Critical Appraisal Checklist for Studies Reporting Prevalence Data (available in https://jbi.global/sites/default/files/2020-08/Checklist_for_Prevalence_Studies.pdf). This tool encompasses nine items about the sampling process, sample size, description of the study subjects, identification/diagnosis of the clinical condition, statistical analysis, and response rate. Adequation for each domain/item will be classified as adequate (YES), inappropriate (NO), absence of clear information to judgment (UNCLEAR), or not applicable (N / A). This procedure will be done by two reviewers, independently, and the judgments will be confronted. Eventual disagreements in the risk of the bias assessment process will be solved by discussion until consensus. The results of this step will be presented in a table, describing the judgment for each domain assessed and their reason(s).

## Discussion

There are few studies that analyzed the deficiency of vitamins of B complex, especially in low- and middle-income countries. Since the B vitamins have an influence on women’s health and knowing that there are no national surveys with synthesized data on the prevalence of these vitamins among women of reproductive age, pregnant women, and lactating women, it is extremely relevant to know the current panorama regarding deficiencies of these vitamins in the female population, which may support the development of strategic actions related to the prevention, treatment, and reduction of this poor nutritional condition. In addition, it can also assist in the development or strengthening of public policies aimed at this group and guide on possible studies needed in the future. Despite the limitations in including only observational studies, we emphasize that this approach only can show a scenario about the rates of the female Brazilian population with B complex deficiencies over the years, allowing the assessment of trends and changes over time, which, when related to events and facts, serve as potential and not definitive causal inferences. The recognition of public health problems, as well as, the efficacy of interventions, most often start from observational studies, as they allow the involvement of a greater number of participants and analysis for a longer period of time.

## Supplementary Information

**Additional file 1.** Strategies used in the Literature Search.

## Data Availability

All data generated or analyzed during this study will be included in the published systematic review article and will also be available upon request.

## Abbreviations

BDTD: Brazilian Digital Library of Theses and Dissertations
BIREME: Latin American and Caribbean Center for Health Sciences Information
EMBASE: Excerpta Medica Database
Hcy: Homocysteine
JBI: Joanna Briggs Institute
MeSH: Medical Subject Headings
PRISMA: Preferred Reporting Items for Systematic reviews and Meta-Analyses
PROSPERO: Prospective Register of Systematic Reviews
SAM: S-adenosylmethionine
Scielo: Scientific Electronic Library Online
WHO: World Health Organization

## References

[1] Darnton-Hill I, Mkparu UC (2015). Micronutrients in pregnancy in low- and middle-income countries. Nutrients.

[2] Black RE, Victora CG, Walker SP, Bhutta ZA, Christian P, de Onis M (2013). Maternal and child undernutrition and overweight in low-income and middle-income countries. Lancet.

[3] Louzada MLDC, Martins APB, Canella DS, Baraldi LG, Levy RB, Claro RM, et al. Impact of ultra-processed foods on micronutrient content in the Brazilian diet. Rev Saude Publica. 2015;49:1–8. 10.1590/S0034-8910.2015049006211 PMC4560336 26270019

[4] Bailey RL, West KP, Black RE (2015). The epidemiology of global micronutrient deficiencies. Ann Nutr Metab.

[5] Duarte APP, Rodrigues PRM, Ferreira MG, Cunha DB, Moreira NF, Sichieri R (2019). Socio-economic and demographic characteristics associated with risk behaviour patterns for chronic non-communicable diseases in Brazil: data from the National Health Survey, 2013. Public Health Nutr.

[6] Park B, Kim J (2016). Oral contraceptive use, micronutrient deficiency, and obesity among premenopausal females in Korea: the necessity of dietary supplements and food intake improvement. PLoS One.

[7] McArthur JO, Tang HM, Petocz P, Samman S (2013). Biological variability and impact of oral contraceptives on vitamins B6, B12 and folate status in women of reproductive age. Nutrients.

[8] Oh C, Keats EC, Bhutta ZA (2020). Vitamin and mineral supplementation during pregnancy on maternal, birth, child health and development outcomes in low-and middle-income countries: a systematic review and meta-analysis. Nutrients.

[9] Kennedy DO (2016). B vitamins and the brain: mechanisms, dose and efficacy—a review. Nutrients.

[10] Oshiro M, Miguita K, Oliveira RAG, Fonseca LKDFS, Barreto OCDO (2007). Riboflavin deficiency in pregnant women in a public institution of São Paulo city, SP, Brasil. Rev Inst Adolfo Lutz.

[11] Molloy AM, Kirke PN, Brody LC, Scott JM, Mills JL (2008). Effects of folate and vitamin B12 deficiencies during pregnancy on fetal, infant, and child development. Food and nutrition bulletin.

[12] George L, Mills JL, Johansson ALV, Nordmark A, Olander B, Granath F (2002). Plasma folate levels and risk of spontaneous abortion. J Am Med Assoc.

[13] Simpson JL, Bailey LB, Pietrzik K, Shane B, Holzgreve W (2010). Micronutrients and women of reproductive potential: required dietary intake and consequences of dietary deficiency or excess. Part i Folate, Vitamin B12, Vitamin B6. J Matern Fetal Neonatal Med.

[14] Jankovic-Karasoulos T, Furness DL, Leemaqz SY, Dekker GA, Grzeskowiak LE, Grieger JA (2021). Maternal folate, one-carbon metabolism and pregnancy outcomes. Matern Child Nutr.

[15] Laanpere M, Altmäe S, Stavreus-Evers A, Nilsson TK, Yngve A, Salumets A (2010). Folate-mediated one-carbon metabolism and its effect on female fertility and pregnancy viability. Nutr Rev.

[16] Škovierová H, Vidomanová E, Mahmood S, Sopková J, Drgová A, Červeňová T (2016). The molecular and cellular effect of homocysteine metabolism imbalance on human health. In J Mol Sci.

[17] Chrysant SG, Chrysant GS (2018). The current status of homocysteine as a risk factor for cardiovascular disease: a mini review. Expert Rev Cardiovasc Ther.

[18] Mujawar SA, Patil VW, Daver RG (2011). Study of serum homocysteine, folic acid and vitamin b(12) in patients with preeclampsia. Indian J Clin Biochem.

[19] Sanchez SE, Zhang C, Malinow MR, Ware-Jauregui S, Larrabure G, Williams MA (2001). Plasma folate, vitamin B12, and homocyst(e)ine concentrations in preeclamptic and normotensive Peruvian women. Am J Epidemiol.

[20] Ronnenberg AG, Goldman MB, Chen D, Aitken IW, Willett WC, Selhub J (2002). Preconception homocysteine and B vitamin status and birth outcomes in Chinese women. Am J Clin Nutr.

[21] Steluti J, Selhub J, Paul L, Reginaldo C, Fisberg RM, Marchioni DML (2017). An overview of folate status in a population-based study from São Paulo, Brazil and the potential impact of 10 years of national folic acid fortification policy. Eur J Clin Nutr.

[22] World Health Organization. Recommendations on wheat and maize flour fortification meeting report: interim consensus statement. Genebra; 2009. 24809114

[23] World Health Organization, Williams AL, van Drongelen W, Lasky RE, Sanderson M, Lai D, et al. Guideline: daily iron and folic acid supplementation in pregnant women, vol. 46. Genebra: World Health Organization; 2012. 23586119

[24] Brazil, Ministry of Health of Brazil, Secretariat of Health Care, Department of Primary Health Care (2013). National Iron Supplementation Program: general conduct manual.

[25] Brazil, Ministry of Health of Brazil, Secretariat of Health Care, Department of Primary Health Care. National Food and Nutrition Policy. Brasilia; 2013. Available from: http://189.28.128.100/dab/docs/portaldab/publicacoes/national_food_nutrition_policy.pdf. Cited 2022 Jun 16.

[26] Brazil, Ministry of Health of Brazil, Secretariat of Health Care. National Policy for Integral Attention to Women’s Health: principles and guidelines. Brasilia; 2009. Available from: https://www.gov.br/mdh/pt-br/navegue-por-temas/politicas-para-mulheres/arquivo/central-de-conteudos/publicacoes/publicacoes/2015/pnaism_pnpm-versaoweb.pdf. Cited 2022 Jun 16.

[27] Brazil, Presidency of the Republic, Secretary of Policies for Women. National Policy Plan for Women. Brasilia; 2013. Available from: https://oig.cepal.org/sites/default/files/brasil_2013_pnpm.pdf. Cited 2022 Jun 16.

[28] Aromataris E, Munn Z, editors. JBI manual for evidence synthesis: JBI; 2020. Available from https://synthesismanual.jbi.global. 10.46658/JBIMES-20-01.

[29] Moher D, Shamseer L, Clarke M, Ghersi D, Liberati A, Petticrew M (2016). Preferred reporting items for systematic review and meta-analysis protocols (PRISMA-P) 2015 statement. Rev Espanola Nutr Hum Diet.

[30] Khabsa M, Elmagarmid A, Ilyas I, Hammady H, Ouzzani M (2016). Learning to identify relevant studies for systematic reviews using random forest and external information. Mach Learn.

[31] Page MJ, McKenzie JE, Bossuyt PM, Boutron I, Hoffmann TC, Mulrow CD, et al. The PRISMA 2020 statement: an updated guideline for reporting systematic reviews. BMJ. 2021;372(n71). Available from: https://www.bmj.com/content/372/bmj.n71. 10.1136/bmj.n71 PMC8005924 33782057

